# *N*,*N*,2,4,6-Penta­methyl­anilinium hexa­fluoro­phosphate–1,4,7,10,13,16-hexa­oxa­cyclo­octa­decane (2/1)

**DOI:** 10.1107/S1600536813033734

**Published:** 2013-12-18

**Authors:** Yi Qi Chang, Yuan Zhang, Huo Lin Lian

**Affiliations:** a Department of Applied Chemistry, Nanjing College of Chemical Technology, Nanjing 210048, People’s Republic of China

## Abstract

In the title compound, 2C_11_H_18_N^+^·2PF_6_^−^·C_12_H_24_O_6_, the 18-crown-6 mol­ecule has crystallographically imposed inversion symmetry. In the crystal, it inter­acts with the cation through weak C—H⋯O hydrogen bonds. The cations and anions are further linked *via* N—H⋯F and C—H⋯F hydrogen bonds, leading to a sandwich structure .

## Related literature   

For background to the development of ferroelectric pure organic or inorganic compounds, see: Haertling (1999 ▶); Homes *et al.* (2001 ▶). For the structure of a related compound, see: Zhang (2013 ▶).
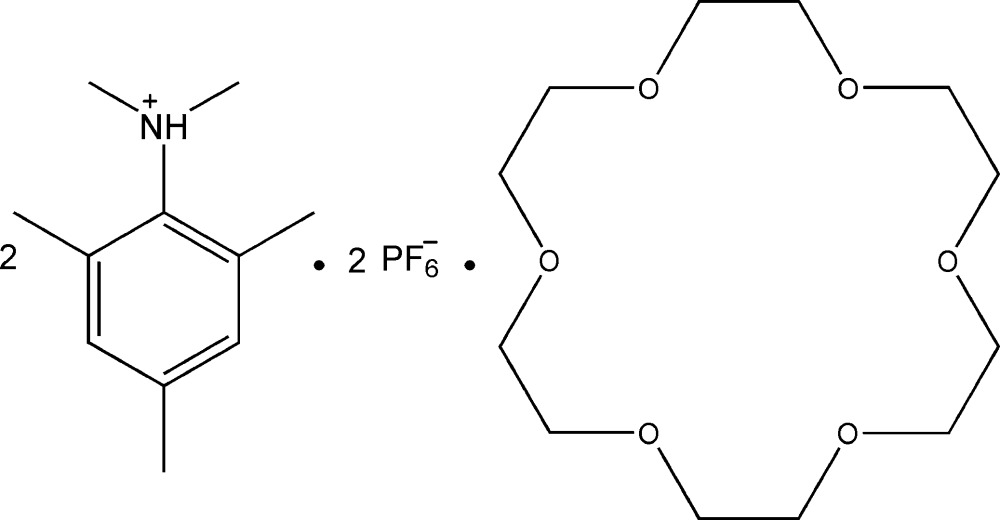


## Experimental   

### 

#### Crystal data  

2C_11_H_18_N^+^·2PF_6_^−^·C_12_H_24_O_6_
*M* *_r_* = 882.78
Monoclinic, 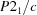

*a* = 8.9122 (18) Å
*b* = 16.775 (3) Å
*c* = 15.136 (3) Å
β = 103.71 (3)°
*V* = 2198.4 (8) Å^3^
*Z* = 2
Mo *K*α radiation
μ = 0.19 mm^−1^
*T* = 293 K
0.40 × 0.30 × 0.20 mm

#### Data collection  

Rigaku Mercury2 (2x2 bin mode) diffractometer
Absorption correction: multi-scan (*CrystalClear*; Rigaku, 2005 ▶) *T*_min_ = 0.832, *T*_max_ = 1.000
18170 measured reflections
3858 independent reflections
2524 reflections with *I* > 2σ(*I*)
*R*_int_ = 0.053

#### Refinement  

*R*[*F* ^2^ > 2σ(*F* ^2^)] = 0.083
*wR*(*F* ^2^) = 0.195
*S* = 1.20
3858 reflections
253 parameters
H-atom parameters constrained
Δρ_max_ = 0.49 e Å^−3^
Δρ_min_ = −0.30 e Å^−3^

### 

Data collection: *CrystalClear* (Rigaku, 2005 ▶); cell refinement: *CrystalClear*; data reduction: *CrystalClear*; program(s) used to solve structure: *SHELXS97* (Sheldrick, 2008 ▶); program(s) used to refine structure: *SHELXL97* (Sheldrick, 2008 ▶); molecular graphics: *SHELXTL* (Sheldrick, 2008 ▶); software used to prepare material for publication: *SHELXTL*.

## Supplementary Material

Crystal structure: contains datablock(s) I, New_Global_Publ_Block. DOI: 10.1107/S1600536813033734/rz5098sup1.cif

Structure factors: contains datablock(s) I. DOI: 10.1107/S1600536813033734/rz5098Isup2.hkl

Supporting information file. DOI: 10.1107/S1600536813033734/rz5098Isup3.cml

Additional supporting information:  crystallographic information; 3D view; checkCIF report

## Figures and Tables

**Table 1:** Hydrogen-bond geometry (Å, °)

*D*—H⋯*A*	*D*—H	H⋯*A*	*D*⋯*A*	*D*—H⋯*A*
N1—H1*C*⋯F4	0.91	2.38	3.077 (5)	134
C16—H16*A*⋯O3^i^	0.96	2.52	3.334 (5)	143
C16—H16*B*⋯O2^ii^	0.96	2.51	3.443 (5)	164
C16—H16*C*⋯O1^i^	0.96	2.57	3.381 (5)	143
C17—H17*B*⋯F4	0.96	2.54	3.122 (6)	119

Symmetry codes: (i) 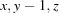; (ii) 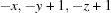.

**Fig. 1.**
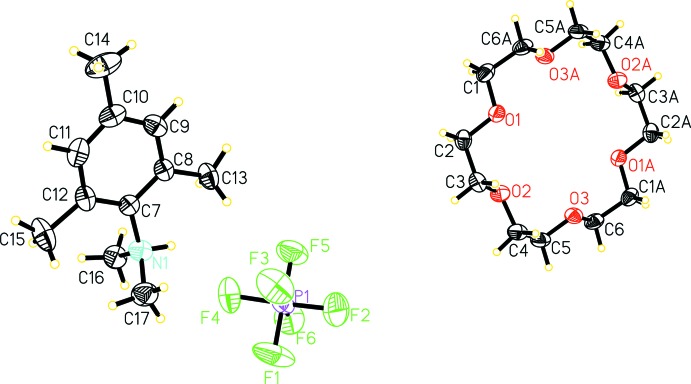
Perspective view of the title compound, showing the displacement ellipsoids drawn at the 30% probability level. Atoms with the suffix A are generated by symmetry code -x, 2-y, 1-z.

**Fig. 2.**
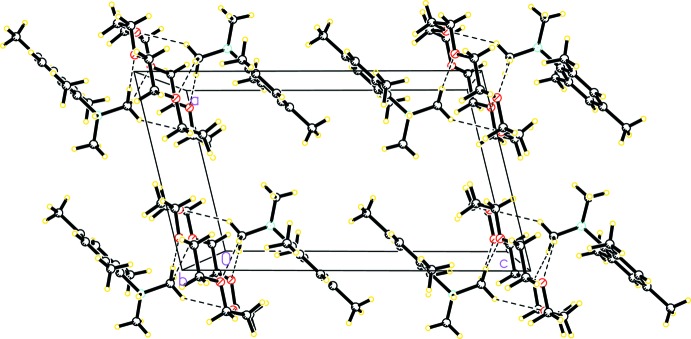
Crystal packing of the title compound viewed along the b axis, showing the hydrogen bonding network (dashed lines).

## supplementary crystallographic information

### 1. Comment

As a continuation of our studies on the development of novel ferroelectric pure organic or inorganic compounds (Haertling *et al.*, 1999; Homes *et al.*, 2001), we investigated the physical properties of the title compound. Recently the crystal structure of the strictly related compound *N*,*N*,2,4,6-pentamethylanilinium hexafluorophosphate was reported by our group (Zhang, 2013). The dielectric constant of the title compound as a function of the temperature indicates that the permittivity is basically temperature-independent (dielectric constant equaling to 4.1 to 6.1), suggesting that this compound should be not a real ferroelectrics or there may be no distinct phase transition occurring within the measured temperature range. Similarly, below the melting point (180°C) of the compound, the dielectric constant as a function of temperature also goes smoothly, and there is no dielectric anomaly observed (dielectric constant equaling to 4.1 to 6.1). Herein, we report the synthesis and crystal structure of the title compound.

The asymmetric unit of the title compound (Fig. 1) consists of one *N*,*N*,2,4,6-pentamethylanilinium cation, one hexafluorophosphate anion and one half of a 1,4,7,10,13,16-hexaoxacyclooctadecane molecule. Bond distances and bond angles are not unusual. In the crystal structure (Fig. 2), the 18-crown-6 molecule interacts with the cation through weak C—H···O hydrogen bonds (Table 1). Cation and anion are further linked *via* N—H···F and C—H···F hydrogen bonds. Dipole–dipole and van der Waals interactions are effective in stabilizing the molecular packing.

### 2. Experimental

A mixture of *N*,*N*,2,4,6-pentamethylbenzenamine (1.36 g, 10 mmol), hexafluorophosphoric acid(1.90 g, 10 mmol) and 1,4,7,10,13,16-hexaoxacyclooctadecane (2.64, 10 mmol) in methanol(30 ml) was stirred until clear. After several days, the title compound was formed and recrystallized from a methanol solution to afford colourless prismatic crystals suitable for X-ray analysis.

### 3. Refinement

H atoms were positioned geometrically and refined using a riding model, with C—H = 0.97 Å, N—H = 0.91 Å, and *U*_iso_(H) = 1.2 *U*_eq_(C, N) or 1.5 *U*_eq_(C) for methyl H atoms

### Crystal data

**Table:**

2C_11_H_18_N^+^·2PF_6_^−^·C_12_H_24_O_6_	*F*(000) = 928
*M**_r_* = 882.78	*D*_x_ = 1.334 Mg m^−^^3^
Monoclinic, *P*2_1_/*c*	Mo *K*α radiation, λ = 0.71073 Å
Hall symbol: -P 2ybc	Cell parameters from 3858 reflections
*a* = 8.9122 (18) Å	θ = 2.6–25.0°
*b* = 16.775 (3) Å	µ = 0.19 mm^−^^1^
*c* = 15.136 (3) Å	*T* = 293 K
β = 103.71 (3)°	Prism, colourless
*V* = 2198.4 (8) Å^3^	0.40 × 0.30 × 0.20 mm
*Z* = 2	

### Data collection

**Table:**

Rigaku Mercury2 (2x2 bin mode) diffractometer	3858 independent reflections
Radiation source: fine-focus sealed tube	2524 reflections with *I* > 2σ(*I*)
Graphite monochromator	*R*_int_ = 0.053
Detector resolution: 13.6612 pixels mm^-1^	θ_max_ = 25.0°, θ_min_ = 3.0°
CCD_Profile_fitting scans	*h* = −10→10
Absorption correction: multi-scan (*CrystalClear*; Rigaku, 2005)	*k* = −19→19
*T*_min_ = 0.832, *T*_max_ = 1.000	*l* = −17→17
18170 measured reflections	

### Refinement

**Table:**

Refinement on *F*^2^	Primary atom site location: structure-invariant direct methods
Least-squares matrix: full	Secondary atom site location: difference Fourier map
*R*[*F*^2^ > 2σ(*F*^2^)] = 0.083	Hydrogen site location: inferred from neighbouring sites
*wR*(*F*^2^) = 0.195	H-atom parameters constrained
*S* = 1.20	*w* = 1/[σ^2^(*F*_o_^2^) + (0.0528*P*)^2^ + 2.*P*] where *P* = (*F*_o_^2^ + 2*F*_c_^2^)/3
3858 reflections	(Δ/σ)_max_ = 0.003
253 parameters	Δρ_max_ = 0.49 e Å^−^^3^
0 restraints	Δρ_min_ = −0.30 e Å^−^^3^

### Special details

**Table:**

Geometry. All e.s.d.'s (except the e.s.d. in the dihedral angle between two l.s. planes) are estimated using the full covariance matrix. The cell e.s.d.'s are taken into account individually in the estimation of e.s.d.'s in distances, angles and torsion angles; correlations between e.s.d.'s in cell parameters are only used when they are defined by crystal symmetry. An approximate (isotropic) treatment of cell e.s.d.'s is used for estimating e.s.d.'s involving l.s. planes.
Refinement. Refinement of *F*^2^ against ALL reflections. The weighted *R*-factor *wR* and goodness of fit *S* are based on *F*^2^, conventional *R*-factors *R* are based on *F*, with *F* set to zero for negative *F*^2^. The threshold expression of *F*^2^ > σ(*F*^2^) is used only for calculating *R*-factors(gt) *etc*. and is not relevant to the choice of reflections for refinement. *R*-factors based on *F*^2^ are statistically about twice as large as those based on *F*, and *R*- factors based on ALL data will be even larger.

### Fractional atomic coordinates and isotropic or equivalent isotropic displacement parameters (Å^2^)

**Table:**

	*x*	*y*	*z*	*U*_iso_*/*U*_eq_	
P1	0.47256 (14)	0.24011 (8)	0.74636 (8)	0.0722 (4)	
F6	0.4805 (5)	0.2762 (2)	0.6529 (2)	0.1280 (12)	
F5	0.3159 (4)	0.2840 (2)	0.7412 (3)	0.1289 (12)	
F4	0.3770 (4)	0.16651 (18)	0.6942 (2)	0.1268 (12)	
F3	0.4597 (5)	0.2030 (2)	0.8388 (2)	0.1461 (15)	
F2	0.5602 (5)	0.3133 (2)	0.7981 (3)	0.1507 (15)	
F1	0.6268 (5)	0.1971 (3)	0.7510 (3)	0.1725 (19)	
O3	0.2823 (3)	1.00490 (16)	0.45178 (17)	0.0589 (7)	
O2	0.1340 (3)	0.85340 (16)	0.45499 (17)	0.0612 (7)	
O1	−0.1059 (3)	0.85014 (16)	0.55077 (17)	0.0609 (7)	
C6	0.2987 (5)	1.0742 (3)	0.4023 (3)	0.0651 (11)	
H6A	0.2276	1.0722	0.3429	0.078*	
H6B	0.4029	1.0771	0.3936	0.078*	
C4	0.2945 (5)	0.8633 (3)	0.4624 (3)	0.0673 (11)	
H4A	0.3358	0.8166	0.4386	0.081*	
H4B	0.3480	0.8696	0.5257	0.081*	
C5	0.3191 (5)	0.9355 (3)	0.4095 (3)	0.0647 (11)	
H5A	0.4261	0.9378	0.4056	0.078*	
H5B	0.2548	0.9321	0.3482	0.078*	
C2	−0.0682 (5)	0.7856 (2)	0.5001 (3)	0.0616 (11)	
H2A	−0.1249	0.7899	0.4371	0.074*	
H2B	−0.0961	0.7357	0.5243	0.074*	
C3	0.1010 (5)	0.7875 (2)	0.5061 (3)	0.0630 (11)	
H3A	0.1573	0.7927	0.5691	0.076*	
H3B	0.1329	0.7384	0.4821	0.076*	
C1	−0.2661 (5)	0.8535 (3)	0.5475 (3)	0.0664 (11)	
H1A	−0.2971	0.8060	0.5751	0.080*	
H1B	−0.3247	0.8560	0.4848	0.080*	
C7	0.0742 (4)	0.0080 (2)	0.7882 (2)	0.0501 (9)	
N1	0.1899 (4)	0.0220 (2)	0.7330 (2)	0.0679 (10)	
H1C	0.1970	0.0761	0.7327	0.081*	
C9	−0.1049 (5)	0.0657 (3)	0.8628 (3)	0.0647 (11)	
H9A	−0.1532	0.1104	0.8797	0.078*	
C12	0.0381 (5)	−0.0678 (2)	0.8125 (3)	0.0616 (11)	
C11	−0.0714 (6)	−0.0732 (3)	0.8639 (3)	0.0726 (13)	
H11A	−0.0971	−0.1235	0.8816	0.087*	
C8	0.0030 (4)	0.0760 (2)	0.8112 (2)	0.0537 (10)	
C10	−0.1433 (5)	−0.0085 (3)	0.8899 (3)	0.0675 (12)	
C13	0.0349 (6)	0.1585 (2)	0.7817 (3)	0.0774 (13)	
H13A	−0.0270	0.1963	0.8049	0.116*	
H13B	0.0099	0.1611	0.7164	0.116*	
H13C	0.1422	0.1709	0.8048	0.116*	
C16	0.1342 (6)	0.0025 (3)	0.6344 (3)	0.0709 (12)	
H16A	0.2140	0.0142	0.6035	0.106*	
H16B	0.0443	0.0338	0.6087	0.106*	
H16C	0.1085	−0.0531	0.6277	0.106*	
C17	0.3506 (5)	−0.0026 (3)	0.7753 (4)	0.0873 (15)	
H17A	0.3774	0.0142	0.8377	0.131*	
H17B	0.4196	0.0217	0.7433	0.131*	
H17C	0.3588	−0.0595	0.7724	0.131*	
C15	0.1046 (7)	−0.1451 (3)	0.7880 (4)	0.1037 (18)	
H15A	0.0592	−0.1889	0.8131	0.156*	
H15B	0.2144	−0.1452	0.8123	0.156*	
H15C	0.0827	−0.1503	0.7230	0.156*	
C14	−0.2611 (6)	−0.0173 (4)	0.9466 (3)	0.109 (2)	
H14A	−0.2989	0.0344	0.9579	0.164*	
H14B	−0.2137	−0.0423	1.0034	0.164*	
H14C	−0.3455	−0.0496	0.9144	0.164*	

### Atomic displacement parameters (Å^2^)

**Table:**

	*U*^11^	*U*^22^	*U*^33^	*U*^12^	*U*^13^	*U*^23^
P1	0.0737 (8)	0.0786 (9)	0.0619 (7)	0.0147 (7)	0.0115 (6)	−0.0153 (6)
F6	0.166 (3)	0.134 (3)	0.090 (2)	−0.004 (2)	0.044 (2)	0.010 (2)
F5	0.105 (2)	0.129 (3)	0.155 (3)	0.044 (2)	0.035 (2)	−0.003 (2)
F4	0.177 (3)	0.088 (2)	0.106 (2)	−0.022 (2)	0.017 (2)	−0.0247 (18)
F3	0.202 (4)	0.166 (4)	0.074 (2)	0.046 (3)	0.041 (2)	0.022 (2)
F2	0.155 (3)	0.151 (3)	0.140 (3)	−0.030 (3)	0.023 (2)	−0.065 (3)
F1	0.126 (3)	0.232 (5)	0.158 (3)	0.101 (3)	0.031 (3)	−0.019 (3)
O3	0.0604 (17)	0.0714 (18)	0.0509 (15)	−0.0010 (14)	0.0253 (13)	−0.0022 (14)
O2	0.0529 (16)	0.0711 (18)	0.0599 (16)	0.0049 (14)	0.0138 (12)	0.0084 (14)
O1	0.0557 (17)	0.0676 (18)	0.0605 (16)	−0.0109 (14)	0.0164 (13)	−0.0133 (14)
C6	0.053 (2)	0.090 (3)	0.057 (2)	−0.012 (2)	0.0249 (19)	0.003 (2)
C4	0.054 (3)	0.079 (3)	0.070 (3)	0.011 (2)	0.019 (2)	−0.001 (2)
C5	0.050 (2)	0.092 (3)	0.058 (2)	0.004 (2)	0.0234 (19)	−0.004 (2)
C2	0.076 (3)	0.054 (2)	0.054 (2)	−0.009 (2)	0.013 (2)	0.0014 (19)
C3	0.075 (3)	0.055 (2)	0.056 (2)	0.007 (2)	0.011 (2)	−0.003 (2)
C1	0.061 (3)	0.074 (3)	0.066 (3)	−0.019 (2)	0.018 (2)	0.000 (2)
C7	0.054 (2)	0.059 (2)	0.0350 (18)	−0.0008 (19)	0.0063 (16)	0.0018 (17)
N1	0.065 (2)	0.083 (3)	0.055 (2)	0.0083 (18)	0.0140 (17)	0.0001 (18)
C9	0.067 (3)	0.078 (3)	0.046 (2)	0.014 (2)	0.006 (2)	−0.008 (2)
C12	0.081 (3)	0.053 (2)	0.046 (2)	0.001 (2)	0.005 (2)	−0.0007 (19)
C11	0.089 (3)	0.069 (3)	0.052 (2)	−0.019 (3)	0.002 (2)	0.008 (2)
C8	0.061 (2)	0.055 (2)	0.040 (2)	0.0030 (19)	0.0023 (18)	−0.0010 (17)
C10	0.052 (2)	0.105 (4)	0.040 (2)	−0.010 (3)	0.0013 (18)	0.006 (2)
C13	0.110 (4)	0.049 (3)	0.075 (3)	0.005 (2)	0.026 (3)	0.001 (2)
C16	0.087 (3)	0.085 (3)	0.045 (2)	−0.004 (2)	0.023 (2)	−0.006 (2)
C17	0.067 (3)	0.090 (4)	0.100 (4)	0.011 (3)	0.010 (3)	0.010 (3)
C15	0.164 (6)	0.057 (3)	0.091 (4)	0.021 (3)	0.032 (4)	0.001 (3)
C14	0.073 (3)	0.187 (6)	0.070 (3)	−0.025 (4)	0.024 (3)	0.011 (3)

### Geometric parameters (Å, º)

**Table:**

P1—F1	1.539 (3)	C7—C8	1.390 (5)
P1—F6	1.556 (3)	C7—N1	1.492 (5)
P1—F3	1.560 (3)	N1—C17	1.482 (5)
P1—F2	1.562 (4)	N1—C16	1.493 (5)
P1—F5	1.564 (3)	N1—H1C	0.9100
P1—F4	1.598 (3)	C9—C10	1.380 (6)
O3—C5	1.404 (5)	C9—C8	1.385 (6)
O3—C6	1.409 (5)	C9—H9A	0.9300
O2—C4	1.418 (4)	C12—C11	1.389 (6)
O2—C3	1.419 (4)	C12—C15	1.507 (6)
O1—C2	1.412 (4)	C11—C10	1.364 (6)
O1—C1	1.418 (4)	C11—H11A	0.9300
C6—C1^i^	1.495 (6)	C8—C13	1.503 (5)
C6—H6A	0.9700	C10—C14	1.512 (6)
C6—H6B	0.9700	C13—H13A	0.9600
C4—C5	1.497 (6)	C13—H13B	0.9600
C4—H4A	0.9700	C13—H13C	0.9600
C4—H4B	0.9700	C16—H16A	0.9600
C5—H5A	0.9700	C16—H16B	0.9600
C5—H5B	0.9700	C16—H16C	0.9600
C2—C3	1.490 (6)	C17—H17A	0.9600
C2—H2A	0.9700	C17—H17B	0.9600
C2—H2B	0.9700	C17—H17C	0.9600
C3—H3A	0.9700	C15—H15A	0.9600
C3—H3B	0.9700	C15—H15B	0.9600
C1—C6^i^	1.495 (6)	C15—H15C	0.9600
C1—H1A	0.9700	C14—H14A	0.9600
C1—H1B	0.9700	C14—H14B	0.9600
C7—C12	1.382 (5)	C14—H14C	0.9600
			
F1—P1—F6	89.5 (2)	C12—C7—C8	122.7 (4)
F1—P1—F3	91.6 (2)	C12—C7—N1	122.0 (4)
F6—P1—F3	178.3 (2)	C8—C7—N1	115.4 (3)
F1—P1—F2	90.6 (3)	C17—N1—C7	116.1 (3)
F6—P1—F2	91.4 (2)	C17—N1—C16	115.4 (4)
F3—P1—F2	89.9 (2)	C7—N1—C16	114.5 (3)
F1—P1—F5	179.7 (2)	C17—N1—H1C	102.6
F6—P1—F5	90.2 (2)	C7—N1—H1C	102.6
F3—P1—F5	88.7 (2)	C16—N1—H1C	102.6
F2—P1—F5	89.4 (2)	C10—C9—C8	122.3 (4)
F1—P1—F4	91.5 (2)	C10—C9—H9A	118.9
F6—P1—F4	89.15 (19)	C8—C9—H9A	118.9
F3—P1—F4	89.5 (2)	C7—C12—C11	116.6 (4)
F2—P1—F4	177.8 (2)	C7—C12—C15	126.6 (4)
F5—P1—F4	88.5 (2)	C11—C12—C15	116.9 (4)
C5—O3—C6	112.2 (3)	C10—C11—C12	123.4 (4)
C4—O2—C3	112.6 (3)	C10—C11—H11A	118.3
C2—O1—C1	112.3 (3)	C12—C11—H11A	118.3
O3—C6—C1^i^	110.0 (3)	C9—C8—C7	117.3 (4)
O3—C6—H6A	109.7	C9—C8—C13	119.2 (4)
C1^i^—C6—H6A	109.7	C7—C8—C13	123.5 (4)
O3—C6—H6B	109.7	C11—C10—C9	117.7 (4)
C1^i^—C6—H6B	109.7	C11—C10—C14	121.5 (5)
H6A—C6—H6B	108.2	C9—C10—C14	120.7 (5)
O2—C4—C5	109.0 (3)	C8—C13—H13A	109.5
O2—C4—H4A	109.9	C8—C13—H13B	109.5
C5—C4—H4A	109.9	H13A—C13—H13B	109.5
O2—C4—H4B	109.9	C8—C13—H13C	109.5
C5—C4—H4B	109.9	H13A—C13—H13C	109.5
H4A—C4—H4B	108.3	H13B—C13—H13C	109.5
O3—C5—C4	110.4 (3)	N1—C16—H16A	109.5
O3—C5—H5A	109.6	N1—C16—H16B	109.5
C4—C5—H5A	109.6	H16A—C16—H16B	109.5
O3—C5—H5B	109.6	N1—C16—H16C	109.5
C4—C5—H5B	109.6	H16A—C16—H16C	109.5
H5A—C5—H5B	108.1	H16B—C16—H16C	109.5
O1—C2—C3	108.6 (3)	N1—C17—H17A	109.5
O1—C2—H2A	110.0	N1—C17—H17B	109.5
C3—C2—H2A	110.0	H17A—C17—H17B	109.5
O1—C2—H2B	110.0	N1—C17—H17C	109.5
C3—C2—H2B	110.0	H17A—C17—H17C	109.5
H2A—C2—H2B	108.4	H17B—C17—H17C	109.5
O2—C3—C2	108.7 (3)	C12—C15—H15A	109.5
O2—C3—H3A	109.9	C12—C15—H15B	109.5
C2—C3—H3A	109.9	H15A—C15—H15B	109.5
O2—C3—H3B	109.9	C12—C15—H15C	109.5
C2—C3—H3B	109.9	H15A—C15—H15C	109.5
H3A—C3—H3B	108.3	H15B—C15—H15C	109.5
O1—C1—C6^i^	109.3 (3)	C10—C14—H14A	109.5
O1—C1—H1A	109.8	C10—C14—H14B	109.5
C6^i^—C1—H1A	109.8	H14A—C14—H14B	109.5
O1—C1—H1B	109.8	C10—C14—H14C	109.5
C6^i^—C1—H1B	109.8	H14A—C14—H14C	109.5
H1A—C1—H1B	108.3	H14B—C14—H14C	109.5

Symmetry code: (i) −*x*, −*y*+2, −*z*+1.

### Hydrogen-bond geometry (Å, º)

**Table:**

*D*—H···*A*	*D*—H	H···*A*	*D*···*A*	*D*—H···*A*
N1—H1*C*···F4	0.91	2.38	3.077 (5)	134
C16—H16*A*···O3^ii^	0.96	2.52	3.334 (5)	143
C16—H16*B*···O2^iii^	0.96	2.51	3.443 (5)	164
C16—H16*C*···O1^ii^	0.96	2.57	3.381 (5)	143
C17—H17*B*···F4	0.96	2.54	3.122 (6)	119

Symmetry codes: (ii) *x*, *y*−1, *z*; (iii) −*x*, −*y*+1, −*z*+1.
