# *N*,*N*,2,4,6-Penta­methyl­anilinium hexa­fluoro­phosphate–1,4,7,10,13,16-hexa­oxa­cyclo­octa­decane (2/1). Corrigendum

**DOI:** 10.1107/S2056989022007125

**Published:** 2022-07-12

**Authors:** Yuan Zhang, Huo Lin Lian, Yi Qi Chang

**Affiliations:** a Department of Applied Chemistry, Nanjing College of Chemical Technology, Nanjing 210048, People’s Republic of China

## Abstract

In the paper by Chang *et al.* [ *Acta Cryst.* (2014), E**70**, o72], there is an error in the order of the authors.

## Abstract 2

Corrigendum to *Acta Cryst.* (2014), E**70**, o72.

The order of the authors in the paper by Chang *et al.* (2014 ▸) is incorrect and should be as given above.

## References

[bb1] Chang, Y. Q., Zhang, Y. & Lian, H. L. (2014). *Acta Cryst.* E**70**, o72. 10.1107/S1600536813033734 PMC3914100 24527005

